# Love at First Taste: Induction of Larval Settlement by Marine Microbes

**DOI:** 10.3390/ijms21030731

**Published:** 2020-01-22

**Authors:** Sergey Dobretsov, Daniel Rittschof

**Affiliations:** 1 Centre of Excellence in Marine Biotechnology, Sultan Qaboos University, Al Khoud 123 P.O. Box 50, Muscat 123, Oman; 2 Department of Marine Science and Fisheries, College of Agricultural and Marine Sciences, Sultan Qaboos University, Al Khoud 123 P.O. Box 34, Muscat 123, Oman; 3 Marine Science and Conservation, Marine Laboratory, Nicholas School, Duke University, 135 Duke Marine Lab Road, Beaufort, NC 28516, USA; ritt@duke.edu

**Keywords:** biofilm, larval settlement, bacteria, diatoms, protozoa, settlement pheromones

## Abstract

Marine biofilms are composed of many species of bacteria, unicellular algae, and protozoa. Biofilms can induce, inhibit, or have no effect on settlement of larvae and spores of algae. In this review, we focus on induction of larval settlement by marine bacteria and unicellular eukaryotes and review publications from 2010 to September 2019. This review provides insights from meta-analysis on what is known about the effect of marine biofilms on larval settlement. Of great interest is the impact of different components of marine biofilms, such as bacteria and diatoms, extracellular polymeric substances, quorum sensing signals, unique inductive compounds, exoenzymes, and structural protein degradation products on larval settlement and metamorphosis. Molecular aspects of larval settlement and impact of climate change are reviewed and, finally, potential areas of future investigations are provided.

## 1. Introduction

Any clean substratum submerged in seawater is quickly colonized by molecules, micro-organisms [1], and propagules [2]. On the surface of the substratum, microbial colonizers form complex biofilms composed of bacteria, archaea, fungi, protozoa, and unicellular microalgae [3]. Marine biofilms are mixed complex microbial communities dominated by bacteria and diatoms (Bacillariophyceae) [4]. Biofilms are three dimensional in structure. Micro-organisms in biofilms are surrounded by a self-produced matrix of extracellular polymeric substances (EPS), composed of polysaccharides, proteins, nucleic acids, and lipids [5]. EPS keep microbial cells together and provide suitable micro-environmental conditions [6]. Marine biofilms are highly dynamic. Changes in temperature, salinity, solar radiation, etc., result in corresponding changes in microbial communities inhabiting biofilms [3]. Additionally, in the presence and absence of macro-organisms micro-organisms interact with each other within a biofilm, which changes microbial densities, biofilm’s species composition, and chemistries in space and time [7]. Products of exoenzymes, part of degradative microbial processes on structural materials, including proteins and proteinaceous glues, are integral to the organization of marine communities [8].

Investigations of marine biofilms, their composition, and dynamics started less than 100 years ago with the pioneering work of Claude Zobell and Ester Allen [9] who observed microbial biofilms and developed culture-dependent methods to isolate and cultivate marine bacteria. This pioneering work on isolation and culturing of bacteria began a fruitful area of research [10]. Culture-dependent methods continue to be intensively used and have been supplemented by development of molecular and metagenomic methods (see reviews [3,4,7,11]). Polymerase chain reaction combined with high throughput next generation sequencing methods allow characterization of non-cultivable and rare microorganisms present in biofilms [12]. Additionally, these methods allow identification of genes and prediction of their functions [3]. Recently developed metabolomic methods are employed to identify metabolites produced by microbes in biofilms in response to environmental stress [13,14]. Combinations of metagenomics, transcriptomics, proteomics, and metabolomics methods enables study of changes in composition of biofilms and chemical compounds produced as well as their functions. Though still in their infancy with respect to analysis and interpretation, these new methods have already demonstrated remarkable potential.

Cell-to-cell communication called quorum sensing (QS) allows bacteria in biofilms to coordinate their adhesion, swarming, luminescence, and production of bioactive compounds [15,16]. Bacteria produce and release small inductive molecules (autoinducers) and respond to them once they reach to a threshold concentration [17,18]. QS autoinducers depend on the density of producing bacteria. Different Gram-positive and Gram-negative bacteria use different QS signals for communication (see reviews [15,16,19]). The best studied QS autoinducers are acyl homoserine lactones (AHLs). Specificity of this autoinducers is related to the length of R-group side chain. Bacteria not only produce QS signals but also inhibit or disrupt QS of other bacteria (see reviews [20,21,22]). Additionally, some marine eukaryotes respond to bacterial QS signals and disrupt them [16]. 

Marine organisms that are sessile as adults routinely have free living planktonic dispersal stages called larvae and sporelings and other kinds of propagules [11]. These are referred to generally as propagules. During the planktonic stage, propagules may or may not, feed, grow, and develop. All propagules disperse some distance in the water column before they settle [23,24]. While some species of marine invertebrates are highly specialized and settle in the particular habitats, other species are generalists and settle and attach to any available substratum [25]. For most propagules, the selected substratum is particularly important for subsequent survival and reproduction. Once larvae or spores attach to the substratum, they metamorphose into juveniles and gather energy growing quickly or slowly into adult organisms. Many macro-fouling organisms involved in fouling of man-made surfaces grow very quickly and are reproductive within weeks of settlement.

The relationship of propagules to the substrate is complex. Microbial biofilms can either induce, inhibit or have no effect on the settlement of marine organisms (see reviews [7,25,26]). Depending on the species, settlement can be rapid, even faster than initial attachment by bacteria and can be triggered by surface energy and organic films [27,28,29,30]. In many cases, specialist larvae require general or specific bacterial films for settlement [25,26,31].

All larvae of specialist species and most larvae of generalist species respond to cues and pheromones produced by symbionts and conspecifics. Differences in a biofilm’s species composition, age, and the density of different species influence the attractiveness of the substratum to larvae through modification of its physical and chemical properties [26,31,32,33].

Biofouling is undesirable growth of micro- and macro-organisms on man-made structures. Biofouling causes huge economic losses to maritime industries, thus development of antifouling solutions that prevent settlement of larvae is of particular importance [34,35,36]. Microbes are so adaptive that usually even antifouling coatings are colonized by bacteria and diatoms [13,37]. 

The process of larval settlement is particularly important for biofouling and aquaculture research [11]. Sessile species, like the barnacle *Amphibalanus* (=*Balanus*) *amphitrite*, the polychaete *Hydroides elegans*, and the bryozoan *Bugula neritina* are global major biofouling species [25]. Larval settlement is important for aquaculture of mussels, oysters, abalone, scallops, and algae. Important to restoration ecology is the rehabilitation of habitat-forming species, like hard corals and oysters, and restoration of the stocks of commercially important shellfish [11]. 

Over the past several decades few reviews about marine biofilms have been published and there are even fewer reviews of larval-biofilm interactions [3,4,7,11,38]. Previous reports show that biofilms can induce or inhibit larval settlement and metamorphosis [7,26,28,39]. 

Since the discovery of natural products inhibitors of biofouling [29,40,41], natural products from marine organisms, including bacteria and diatoms, that inhibit biofouling have been reviewed [7,42,43,44,45,46]. Several reviews analyzed chemical ecology and natural products of microbes, invertebrates, vertebrates, and plants [47,48,49,50].

Hadfield and Paul [51] provided a comprehensive review of induction of larval settlement of invertebrates from 10 phyla by biofilms. Subsequent reviews focused either on biofouling and commercially important species [11,25] or particular processes, like quorum sensing and its inhibition [16,22]. Molecular mechanisms of larval settlement and inhibition were recently reviewed as well [52,53,54,55]. The development of novel omic-methods (metagenomics, metabolomics, transcriptomics, proteomics) resulted in many new findings in larval settlement and attachment. Thus, it is timely to summarize findings on induction of larval settlement by marine biofilms. 

The main aim of this publication is to review recent publications on the induction of propagule settlement of macro-organisms by microbial biofilms published from January 2010 to September 2019. For earlier scientific publications readers should refer to above cited reviews and papers on this subject. Of great interest of this review is the impact of different components of marine biofilms, such as exoenzymes, EPS, bacteria and diatoms, QS autoinducers on larval settlement and metamorphosis, and unique inductive compounds from marine microorganisms. Molecular aspects of larval settlement are reviewed and, finally, future perspectives, and conclusions are provided. Additionally, the effect of factors associated with climate change on biofilms and larval settlement is discussed. Our ultimate goal is to begin to provide a coherent picture of the range of critical roles that microbes play in the development of marine communities.

## 2. Meta-Analysis of Recent Publications

**Figure 1:**
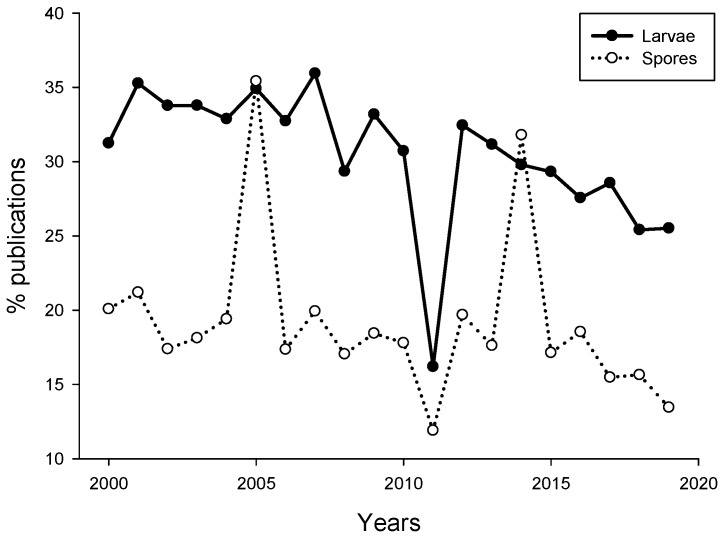
Percent of publications dealing with settlement of invertebrate larvae (black circle) and algal spores (open circle) and microbes. The search was performed using Google Scholar for the period of 2000–2019. The search keywords were “marine microbe” and “larva” or “marine microbe” and “spore”.

Analysis of publications suggested that the number of papers dealing with induction or inhibition of propagules settlement published for the last 19 years remains quite low (469). The percentage of publications dealing with larval settlement in response to biofilms did not exceed 35%, while the percentage of algal spore publications even lower (~21%, Figure 1). There was a sudden drop in the percentage of larval settlement and algal spore publications in 2011.

The reason for that drop in the percentage of publications is not clear. Overall, the percentage of studies investigating propagule settlement decreased from 2000 to 2019. This may be related to the decrease of biofouling-related publications in recent years due to decreased funding opportunities in basic research on marine larvae and increased funding for basic and applied studies related to biofouling management [56].

**Figure 2:**
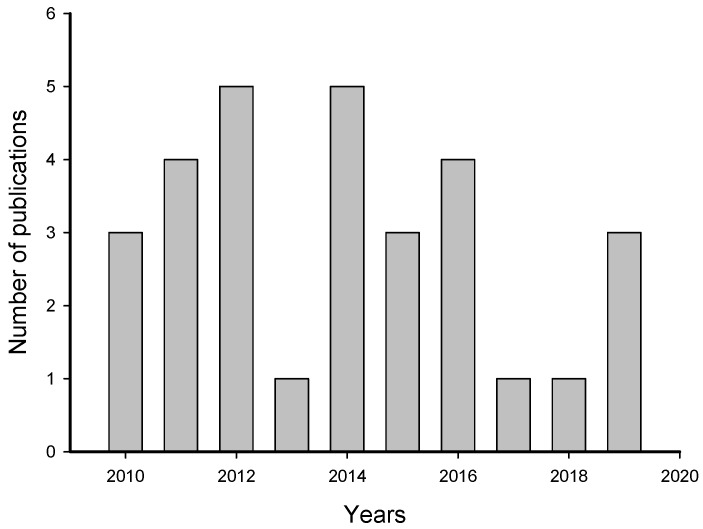
Number of publications that examined the induction of larval settlement by marine biofilms. The publications include from January 2010 to September 2019. This figure only includes the publications that met all inclusion criteria.

The systematic review of 469 publications by the PRISMA (Preferred Reporting Items for Systematic Reviews and Meta-Analyses) method resulted, after citation screening, in 31 articles about induction of larval settlement by biofilms (Table S1). During the screening, duplicates and articles not related to the topic as well as reviews were eliminated. The number of publications that met our criteria increased from 2010 to 2012 and decreased from 2014 to 2019 (Figure 2). The highest numbers of publications (5) about induction of larval settlement by biofilms were published in 2012 and 2014 and the lowest number (1) was published in 2013, 2017, and 2018. Overall, the average rate of publication was 3.1 papers per year.

**Figure 3:**
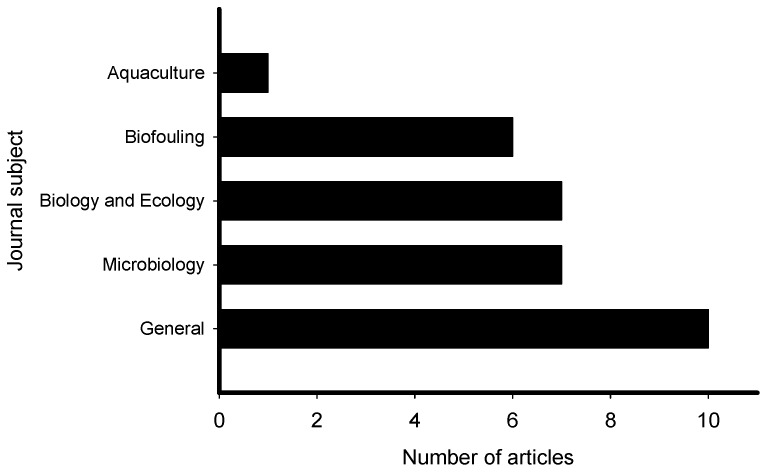
Number of articles that examined the induction of larval settlement by marine biofilms from January 2010 to September 2019 according to the subject area. This figure only includes the publications that met all inclusion criteria.

**Figure 4:**
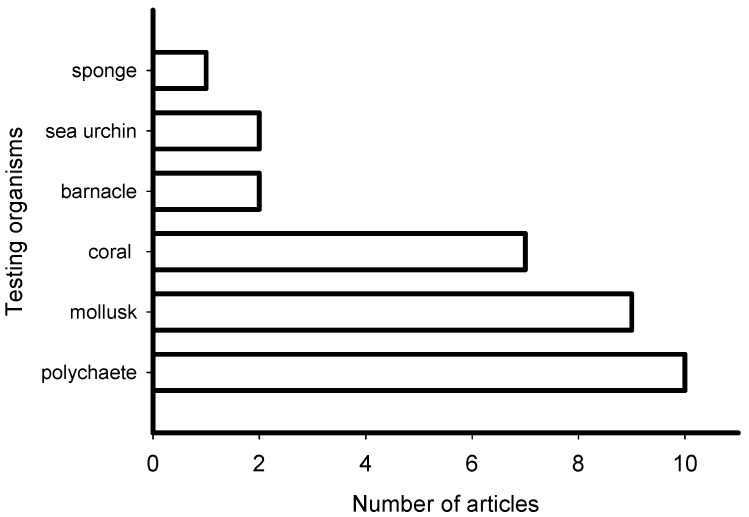
Number of articles that examined the induction of larval settlement by marine biofilms from January 2010 to September 2019 according to the group of larvae. This figure only includes the publications that met all inclusion criteria.

The reviewed articles on induction of larval settlement by marine biofilms were published in journals that represented general interest (e.g., PLoS ONE, Science, Scientific Reports), microbiology (ISME Journal, Microbial Ecology), marine ecology and biology (Marine Ecology Progress Series), biofouling, and aquaculture fields (Figure 3). The highest number of articles (10) was published in general field journals with open access and the lowest number (1) was published in an aquaculture journal. However, though low in number the collective group of papers was represented in very high impact factor journals. All 31 articles reported induction of larval settlement by bacteria or biofilms (Table S1). Not any diatom-related study reporting induction of larval settlement was published in the last 10 years. Fifteen of 31 articles reported effects of monospecies films of bacteria, while 16 of 31 reported effects of multispecies biofilms. The highest number of articles reported induction of polychaete settlement (mostly *Hydroides elegans*) followed by mussel (mostly *Mytilus* spp.) larval settlement (Figure 4). Induction of coral planulae settlement by bacteria and biofilms is represented by seven articles. One article was published on induction of sponge larvae settlement by bacterial biofilms.

Most publications focused on species with high economic significance, either because they can cause a significant biofouling problem (e.g., barnacles and polychaetes) or because they are commercially exploited (e.g., mussels and corals). By in large, our conclusion on the topic of publications is due to the decrease in availability of funding and need to target focused bacteria-larvae interactions of specialist settlers, like *Hydroides elegans* in order to be competitive for that limited funding. Support for this view is limited to observations by Holm [56] on productivity of publications on the best-studied model organisms for fouling management, barnacles, as compared to other model organisms with global distribution such as calcareous tube worms and bushy bryozoans. Holm showed reports of studies using barnacles are outstripping other biofouling models due to a focus on barnacles as globally important biofoulers. Because biofouling is a major driver of a major source of funding, when several funding agencies switched from biological solutions for biofouling management materials science solutions both micro- and macro-fouling research were less supported. These changes contributed to the decrease of reports of bacterial induction of settlement of generalist species, especially of less mature models such as bryozoans and ascidians that have highly variable responses to microbial films, are under-represented in the last decade. In this field, part of the changes represents a shift in focus from interaction with bacteria to studies of biological glues, novel materials, and less environmentally damaging forms of fouling management.

## 3. Induction of Larval Settlement by Bacteria and Biofilms

### 3.1. Multispecies Biofilms and Macrofouler Settlement

From 2010 to 2019, the majority of researchers studied the effect of multispecies biofilms on larval settlement of polychaetes, mollusks, and corals (Figure 4; Table S1). Researchers observed highly diverse bacterial biofilms developed on different substrata in intertidal and subtidal zones [57]. The composition of these biofilms varied with season and tidal level [57], as well as with biofilm age [58]. Additionally, biofilm age and the type of substratum alter metabolites produced by microorganisms (biofilm’s chemical profile) [59].

**Figure 5:**
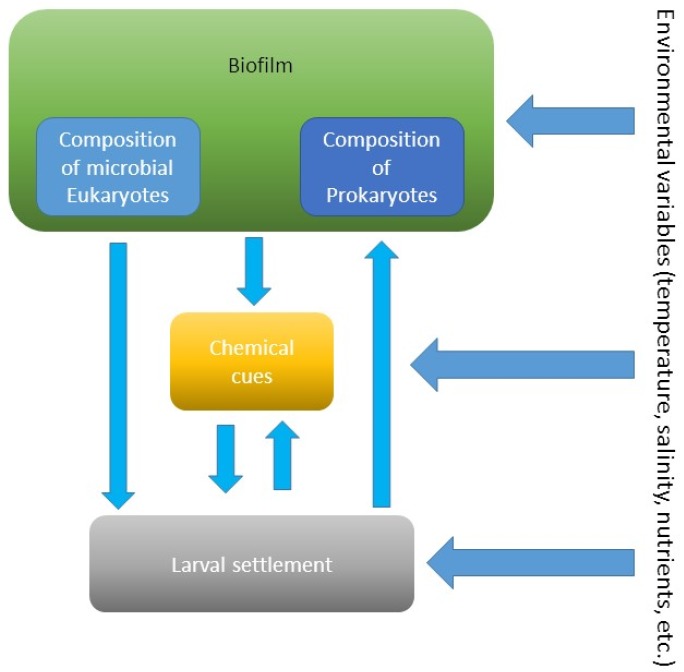
Interactions between biofilms, their chemical compounds, and larvae of invertebrates at variable environmental conditions.

The studies demonstrated that age-dependent composition of biofilms determines settlement of invertebrate larvae (Figure 5). For example, older biofilms induced higher larval settlement of *Mytilus coruscus* compared to young biofilms [58]. The authors concluded that the change in bacterial community composition could explain the change in biofilm’s attractiveness. Similar findings were observed for larval settlement of the bivalve *Mytilus edulis* [60]. Moreover, the inductiveness of biofilms for the filter-feeding bivalve was higher in the absence of food (phytoplankton). This suggests that the influence of composition of biofilms could be more critical larval settlement when nutrient resources are absent or limited (Figure 5).

Larvae of the sponge *Rhopaloeides odorabile* settle more on biofilms developed over longer periods and at higher temperatures [61]. This suggests that environmental variables, such as temperature, affect the composition and probably metabolites of biofilms, which, in turn, affect settlement of larvae (Figure 5). During settlement, larvae of the sea urchin *Heliocidaris erythrogramma* respond to the specific biofilm composition on the surface of macroalgae [62]. In contrast, another sea urchin *Holopneustes purpurascens* does not respond to epibiotic biofilms [62]. 

Settlement of the barnacle *Amphibalanus (=Balanus) amphitrite* on biofilms developed in the intertidal zone was higher than on biofilms developed in the subtidal zone, which correlated with the presence of Proteobacteria and Cyanobacteria species [57]. While composition of biofilms developed at inshore and offshore sites was different, larvae of the specialist species *H. elegans* indiscriminately settled on all biofilms [63]. Settlement of *H. elegans* was strongly correlated with bacterial abundances in biofilms, which confirmed previous findings [64,65]. Since the density of bacteria and their exopolymers usually increases over time, it is not surprising that extracts of older biofilms induced higher settlement of *H. elegans* than the younger ones [59].

The presence of pollutants, like heavy metals, can change microbial composition of biofilms and, in turn, can affect larval settlement (Figure 5). The settlement of the polychaete *H. elegans* on biofilms developed in the presence of copper was lower compared to pristine biofilms [66]. Moreover, the inductiveness of young biofilms was more easily altered by the presence of copper than that of older biofilms. Bao et al. [66] suggest that these changes were due to changes in biofilm composition and production of microbial chemical compounds.

### 3.2. Monospecies Bacterial Films and Macrofouler Settlement

Most natural biofilms are complex communities which include Prokaryotes and unicellular Eukaryotes. Because this complexity cannot be duplicated in controlled conditions most researchers work with cultivable bacteria in monospecies biofilms in the laboratory (Table S1). While such biofilms never exist in the marine environment, they are useful tools for investigation of mechanisms of larval settlement. Cell cultures of α-Proteobacterial strain, *Roseivivax* sp. 46E8, significantly increased larval settlement in *Porites astreoides* only during active growth phase [67]. Treatment of monospecies biofilms of *Shewanella* sp. 1 with antibiotics, formalin, and ultra-violate radiation significantly reduce settlement of *Mytilus coruscus* [68]. This indicates that live bacteria produce inductive cues. While the red colored bacterium *Pseudoalteromonas* sp. SF57 inhibits larval settlement of the polychaete *H. elegans*, the white mutant WM1 of this bacterium is inductive [69]. Analysis of the disrupted genes suggests that the type II secretion pathway, the LysR transcriptional regulator, NAD(P)-binding proteins, exonuclease, pyruvate metabolism, flagella assembly, and cell membrane processes play a role in the regulation of pigmentation and inductiveness of *Pseudoalteromonas* sp. [69].

**Figure 6:**
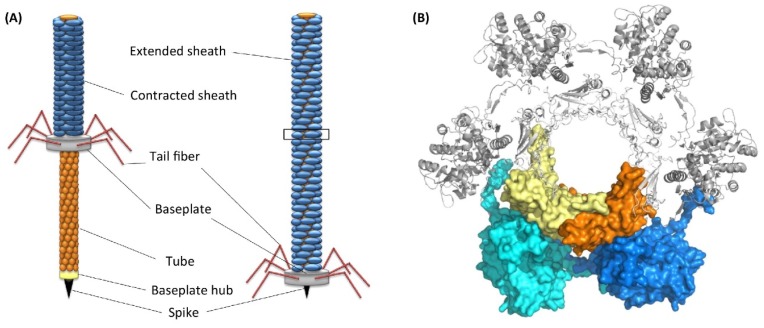
(**A**) Structure of tailocins of bacteria. (**B**) Top view of a transverse section of tail tube. Reproduced with permission from Chequire and De Mot [70].

The marine bacterium *Pseudoalteromonas luteoviolacea* produces phage tail-like structures (tailocins) that comprise of contractile structures with outward-facing baseplates linked by tail fibers and a hexagonal sheath [70] (Figure 6). These structures have bacteriocin activities. It has been demonstrated using bacterial mutants that tailocins or metamorphosis-associated contractile structures (MACs) of *P. luteoviolacea* trigger metamorphosis of *H. elegans* [71,72]. Sequencing of the genome and transcripts of *H. elegans* before and after larval settlement revealed that MACs induce the regulation of groups of genes important for tissue remodeling, innate immunity, and mitogen-activated protein kinase signaling [71].

The following sequence of bacteria-induced metamorphic events was proposed. First, MACs induce larval settlement. Second, MACs encoded by a specific locus in *P. luteoviolacea* initiate larval cilia loss and activate metamorphosis-associated transcription. Finally, signaling through mitogen-activated protein kinase pathways alters gene expression and leads to initiation of metamorphosis [71]. A subsequent study investigated the presence of tailocins in other inductive species of bacteria, such as *Cellulophaga lytica*, *Bacillus aquimaris*, and *Staphylococcus warneri* [73]. Using electron microscopy and genomic analysis the authors found only *P. luteoviolacea* has tailocins. This suggests that tailocins are not universal inducers across inductive bacterial species.

Another study investigated genes of the marine bacterium *P. luteoviolacea* important for larval settlement of *H. elegans* [74]. Genes *ORF1*, *ORF2*, and *ORF4* absent in non-inductive strain of *P. luteoviolacea* were necessary for induction of larval settlement. This is one of the first studies that identified bacterial genes required for induction of settlement and metamorphosis of a marine invertebrate. Future studies are needed in order to determine if these genes are present and active in other inductive bacteria and to identify gene functions.

## 4. Microbial Eukaryotes and Larval Settlement

Information on effects of microbial eukaryotes on larval settlement is limited (Table S1). Diatoms (Bacilariophytae) induce larval settlement of the polychaete *H. elegans* in laboratory experiments [75]. Lam et.al. [76] reported that carbohydrates not proteins from EPS of the diatoms *Achnanthes* sp. and *Nitzschia constricta* induced settlement of *H. elegans*. Metamorphosis of the abalone *Haliotis asinina* was significantly higher on biofilms of *Navicula* compare to unfilmed surfaces [77]. Similarly, settlement of the mussel *Argopecten purpuratus* was higher on natural diatom biofilms [78]. Cyprids of *Amphibalanus* (*=Balanus*) *amphitrite* predominantly settled on biofilms of *Cocconeis* sp. and *Navicula* *ramosissima* [79]. Higher densities of diatoms triggered higher settlement rate of the barnacle. Larval attachment of the bryozoan *Bugula neritina* correlated with the density of diatoms in biofilms [80]. Dahms et al. [81] demonstrated that different diatom species affect *B. neritina* settlement in different ways. The highest percentage of settlement of larvae of *B. nertina* was mediated by biofilms of *Achnanthes* sp., *Amphora cofeaeformis*, *Amphora tenerrima*, and *Nitzschia constricta*, while the lowest settlement was observed on *Nitzschia frustulum* biofilms. When low inductive and highly inductive diatom strains were combined together in a multispecies biofilm, their resulting activity was always highly inductive for *B. neritina* [81]. Thus, responses to multispecies and monospecies biofilms are different. There have been no publications reporting induction of larval settlement by diatoms from 2010 to 2019 (Table S1). While flagellates are the third largest group in marine biofilms [82], there is no evidence that this group can induce or inhibit larval settlement of invertebrate larvae.

Ciliates and other Protista are a ubiquitous component of biofilms [82]. However, only one study reported impacts of ciliates on settlement of larvae of invertebrates (Table S1). Watson et al. [83] investigated the effect of four different species of planktonic and vagile ciliates on the settlement of the polychaete *Galeolaria caespotisa*. Compared to control, settlement was significantly reduced in the presence of three species of ciliates: *Amphisiella* sp., *Euplotes minuta*, and *Uronema marinum*. Larval settlement was inhibited in the physical presence of ciliates but not with just their metabolites. Additionally, ciliates changed the distribution of bacteria in biofilms indicating that ciliates can control settlement indirectly [83].

## 5. Bioactive Compounds from Biofilms Inducing Settlement and Metamorphosis

### 5.1. Bacterial Chemical Signals

**Figure 7:**
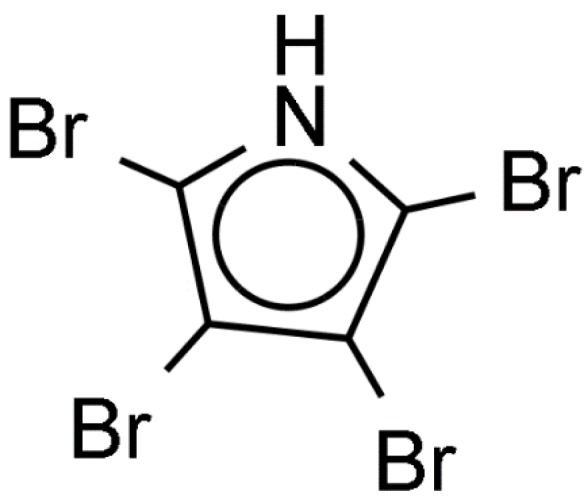
Tetrabromopyrrole produced by *Pseudomonas* bacteria inducing metamorphosis of coral larvae.

Review of publications dealing with induction of larval settlement and metamorphosis by biofilms showed a limited number of studies that identified chemical inducers produced by microorganisms (Table S1). The larvae of the acropoid coral *Acropora millepora* metamorphose in the presence of coralline algae *Neogoniolithon fosliei* and *Hydrolithon onkodes* [84]. *Pseudoalteromonas* bacteria isolated from coralline algae induce metamorphosis of larvae of the acropoid coral *Acropora millepora*. The bacterial metamorphosis cue was identified as tetrabromopyrrole (TBP, Figure 7). While other *Pseudomonas* species produce brominated compounds, only TBP induced almost 100% of larval metamorphosis without attachment [84]. Another study conducted with *Pseudoalteromonas* sp. PS5 associated with coralline algae showed that this strain also produces TBP [85]. TBP induced settlement of several species of corals, such as *Porites astreoides*, *Orbicella (Montastraea) franksi*, and *Acropora palmata*. The effect of TBP binding protein on gene expression of larvae *A. millepora* was studied using reverse-transcriptase quantitative PCR (RT-qPCR) [86]. TBP-containing bacterial extract changed expression of 24 out of 42 genes of interest. Expression of genes was concentration dependent. At the threshold TBP concentration only 14 of these genes were significantly regulated [86]. *Amgalaxin-like-1* genes were upregulated and the *SCRiP* genes were down regulated during larval metamorphosis. Additionally, lectin encoding genes were either not affected or downregulated, suggesting that lectins are not involved in *A. millepora* larval metamorphosis but probably used in larval substrate exploration. This in accordance with previous findings that suggested that lectins, like concanavalin A of larvae *Neodexiospira braziliensis* are involved in larval recognition of biofilmed substrata through specific ‘lock-and-key’ binding with polysaccharides of the bacterial film [87].

### 5.2. Quorum Sensing and Settlement

**Figure 8:**
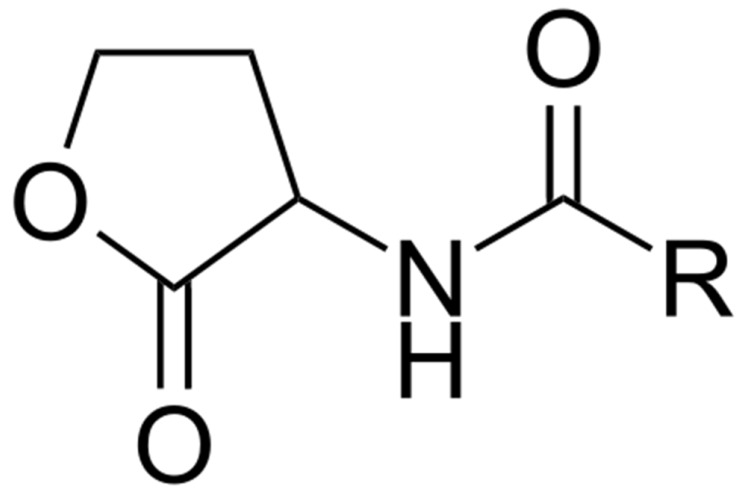
Generalized structure of acyl homoserine lactones (AHLs). R—fatty acid acyl chain with number of carbons from 4 to 18.

Although the first QS compound named acyl homoserine lactone (AHL, Figure 8) was isolated from the marine bacterium *Vibrio fisheri* associated with the light organ of the squid *Euprymna scolopes* [88], information concerning the presence of QS molecules in the marine environment is scarce [15]. AHL QS signals were found predominantly in *Vibrio* species and in the Roseobacter clade. The production of AHLs by bacteria colonizing different marine habitats—such as marine snow [89], marine sponges [90,91], corals [92], and dinoflagellates [93]—have been reported. N-dodecanoyl-L-homoserine lactone (C12-HSL) was found in marine tropical subtidal biofilms [94]. These findings suggest that QS signals are present in biofilms and produced by bacteria in vivo.

QS autoinducer signals produced by bacteria can work as inter-kingdom signals (Table 1). For example, zoospores of the green alga *Ulva* (=*Enteroporpha*) sp. use AHLs produced by *Vibrio anguillarum* to attach [95,96]. Similarly, the epiphytic bacteria *Sulfitobacter* spp. and *Shewanella* spp. associated with the alga *Ulva linza* produce AHLs and these QS signals increased zoospore germination and growth [97]. AHLs produced by epibiotic bacteria associated with *Gracilaria dura* and *Acrochaetium* sp. induce algal spore liberation [98] (Table 1). Interestingly, unsaturated long chain hydrocarbons and dibutylphthalates also induce settlement in *U. linza* [99]. 

Not only macro-algae respond to bacterial QS signals. AHL producing bacterial strains *Vibrio anguillarum*, *Aeromonas hydrophila*, and *Sulfitobacter* sp. induce larval settlement of barnacle larvae *Balanus improvises* [100] (Table 1). Mutants of bacterial strains that do not produce AHLs do not induce barnacle larval settlement. Additionally, synthetic AHLs at concentrations similar to those found within natural biofilms resulted in increased barnacle larval settlement. Similarly, larvae of the polychaete *Hydroides elegans* stop swimming and start crawling on the bottom when exposed to synthetic AHLs or biofilms producing AHLs [94]. These publications suggested that QS signals produced by bacteria could control larval and spore settlement as well as spore germination. There is no clear evidence that eukaryotes evolved with bacteria to sense QS signals and there is no known direct benefit from such communication to the bacteria. 

## 6. Molecular Aspects of Induction of Settlement 

Expanding perspective from consortia communication and tapping into that communication as described above, one can move to the ecosystem level. Ecosystems can be considered as complex symbiotic assemblages. Within estuarine ecosystems, three examples of a scale that provides a tractable experimental unit are clam beds, mussel patches, and oyster reefs. Clam beds, mussels, and oyster reefs are examples of what was classically described in the 1950s and 1960s as gregarious settlement [101,102,103,104,105]. Gregarious settlement is a complex process that involves flows, textures, and species-specific settlement cues. However, unlike QS cues, these settlement cues are much more primitive and less well understood. At least some cues are based upon degradation of structural protein substrates. The best understood of which are proteins degraded by exoenzymes [106].

Oyster reefs are interesting because the biological habitat generated extends off the substrate, undergoes succession and many ecological details are well studied. Oyster reef communities are organized and informed by the actions of symbiotic bacteria that are known mainly through the products of their exo-enzymatic activity on host structural proteins. All this biochemistry provides communication between the nodes of this complex community [8,101,105,107,108,109].

Rather than being based on production and secretion of signal molecules, larvae of gregarious settlers—like oysters—recognize species-specific degradative products that result from actions of exoenzymes on structural proteins and biological glues. The best studied of these settlement pheromones are those associated with gregarious settlement of barnacles and oysters. Though in separate phyla, settlement pheromones can be mimicked by the same pure synthetic and natural neutral amino acids, basic carboxyl terminal amino acid, tripeptides glycil-glycil-arginine that can be generated by the action of trypsin-like serine proteases on proteins found in periostraca, in the organic matrix of shells and by degradation of natural glues [8]. 

The basic structure of an oyster reef is organized by bacterial exoenzymes that produce oyster and barnacle settlement pheromones from barnacle and oyster structural proteins. That not all exoenzymes originate from microbes reflects the complexity and interdependence of complex symbiotic assemblages [8]. The settlement pheromones, in some cases in concert with specific bacteria [114,115], result in gregarious settlement of planktonic larvae. Growth of gregariously settled individuals results in the structure of the reef [108]. Gregarious settlement is important and often essential for reproduction [116]. 

Bacteria-barnacle interactions can at one level be negative [117,118,119]. Settling barnacles kill the bacteria on the surface they are gluing to by releasing reactive oxygen species, antibacterial peptides and activating enzyme cascades associated with the innate immune response [117,120,121]. Similarly, large mobile predators, like blue crabs, use enzymes to remove biofouling from places they cannot scratch, their gills, and their brooded eggs [117]. However, at another level, these processes produce species specific settlement cues and pheromones. Bacteria thrive on cured glues [122] by digesting them with proteolytic enzymes including serine proteinases. Enzyme generated peptides directly impact oysters and barnacles as well as other members of the community. 

In the crabs, both fouling and cleanliness come a cost because they both generate body odors. Prey detect the odors of stone crabs and blue crabs, and avoid contact by fleeing or ‘hiding’ by stopping ventilation (clamming up) while the crab is in the immediate vicinity. In the reefs, body odors enable individual recognition. Snapping shrimp that live in pairs recognize their mates [123] and shrimp that have lost an aggressive encounter with another shrimp recognize the winner by its odor [124].

It is fascinating that barnacle settlement pheromones also attract predatory snails that eat barnacles and oysters and other sessile organisms [125,126]. Predatory snails have tapped into an essential sexual communication system for oysters and barnacles [125]. It is also likely that a parasitic symbiotic crab that lives in oysters uses these cues as do the intermediate hosts of trematode parasites that cycle through oysters fish and birds.

Over the last 15 years, understanding of the roles of exoenzyme actions is expanding [106,117,118,119,120,121,122,125,126,127,128]. However, this is just the tip of the iceberg. New shotgun proteomics technologies and microbiome technologies provide huge potential for study of this most basic of positive interactions between microbes and gregarious macro-organisms. 

## 7. Impact of Climate Change on Biofilms and Larval Settlement

Release of anthropogenic CO_2_ in the atmosphere increases temperatures of our planet [129]. This increases sea surface temperatures of the oceans and leads to ocean acidification [130]. Such changes will have a direct and indirect impact on biofilms, chemical cues, and larval settlement [131]. In several studies, the impact of increased sea temperature and acidification on different species of biofouling organisms has been studied. It is predicted that acidification will impact aragonite and magnesium calcite producers—such as coralline algae, corals, mussels, barnacles, and some bryozoans [132,133,134,135]—and will increase abundances of soft-bodied organisms [136]. At the same time, information about the impact of climate change on biofilms and larvae of invertebrates is limited [131].

Climate change can alter the structure of microbial communities, which, in turn, can have impact on the structure of macro-fouling communities [60]. Microbial communities developed at 23 °C and 30 °C had different structure from those developed at 16 °C [137]. Settlement of larvae of barnacles of *Amphibalanus* (*=Balanus*) *amphitrite* and *Balanus trigonus* on biofilms developed at high temperature was different from settlement on biofilms developed at low temperatures. Similarly, only biofilms developed at elevated temperatures stimulated sponge larval settlement [60].

Acidification also affects microbial communities. For example, seawater acidification (a decrease of 0.2 pH units) over 100 days decreased the density of diatoms, which in turn, increased densities of sponges, tunicates, and decreased the density of spirorbids [136]. These examples show that factors associated with climate change directly affect the composition and densities of microorganisms in biofilms and indirectly reduce or enhance larval settlement of macro-fouling species.

## 8. Future Studies

In general, there was a substantial amount of work on the roles of microbes and larval settlement from the 1980s until the first decade of the 2000s and this work is comprehensively reviewed by Hadfield and Paul [51]. Most, if not all, of this work focused on microbial films on inert surfaces and features of the bacteria, like age of film, and components of the film, like EPS. The majority of studies are about induction of larval settlement by bacteria. The new *omic* techniques—such as metagenomics, metabolomics, proteomics, and transcriptomics—allow to characterize microbes in biofilms, identifying metabolites produced and genes responsible for their production [11,12,13,14,86]. In the future, the development of such techniques and the lower cost of such methods will result in an increase of molecular studies of microbe–larva interactions. This will enhance our understanding of receptors, genes, and pathways responsible for larval settlement and metamorphosis. 

The future of ‘omics’ technologies is extension of these technologies to interactions between larvae and bacteria living on complex biological substrates like macroalgae, shells, biological glues, and wood. This aspect is especially important for understanding gregarious settlement, which often depends on interactions of exoenzymes from bacteria and symbionts on structural components during feeding and cleaning. Although there is ecological evidence that there is induction of especially gregarious larval settlement by less investigated groups of microorganisms—like diatoms, ciliates, and flagellates—the technology is now available to study these phenomena.

Specific bacterial–larval interactions are productive and worthy of continued investigation. Another area understudied by modern techniques is settlement due to constitutive and induced exoenzymes and their products should be a fruitful area of research especially with microbiome technology, proteomic and metabolomic and shotgun sequencing techniques [52,138]. The majority of bacteria secrete enzymes when they feed on biological substrates. We postulate products that escape consumption may be central to the organization of marine communities [8]. Degradation products of structural proteins generated by serine proteases and hydrolysis of complex amino sugars are the only examples to date [106] and more future research is required. 

## 9. Conclusions 

Analysis of published literature suggested that induction of larval settlement by marine microbes is by three major routes: (1) release of inductive molecules including those involved in quorum sensing from established biofilms and consortia; (2) release of exoenzymes during microbial feeding generating degradation products that act as settlement cues and pheromones; (3) inductive molecules and even physical viral-like structures, like tailocins found within bacteria. In the last decade, a relatively small number of very high impact reports significantly advanced the field of microbe macro-fouler interactions to include genes involved in settlement and metamorphosis, specific groups of inductive microbes, and physically unique induction mechanisms. Most recent advances employed sophisticated and new molecular biology techniques. These reports are exciting and at the same time humbling in that they support our contention that understanding of the roles of bacterial induction of settlement are in their research infancy. The future in this area is in communities, large symbiotic assemblages, and the impact of environmental change due to human impacts on complex ecosystems. 

## Figures and Tables

**Table 1:** Quorum sensing (QS) compounds production and their effect on eukaryotes

Target Species	Source of QS	QS Molecule	Mechanism	Reference
**Algae**				
*Ulva (Enteromorpha)* sp.	*Vibrio anguillarum*	3-oxo-C12-HSL C6-HSL	Attachment of spores	[95,96,110]
*Ulva* sp.	*Schewanella* sp.	C4-HSL, C6HSL, C12-HSL	Attachment of spores	[111]
*Ulva linza*	Different species	C12-HSL	Germination of spores	[97]
*Gracilaria dura*	Epibiotic bacteria	C6HSL	Spore liberation	[112]
*Scrippsiella trochoidea*	Epibiotic bacteria	?	Growth	[113]
*Acrochaetium*	Epibiotic bacteria	C4HSL	Spore liberation	[98]
**Polychaete**				
*Hydroides elegans*	Biofilms	C6-HSL, C12-HSL, 3-oxo-C8-HLS	Settlement behavior	[94]
**Barnacle**				
*Balanus improvisus*	*Vibrio anguillarum, Aeromonas hydrophila, Sulfitobacter* sp.	C8-HSL, 3-oxo-C10-HSL, C12-HSL	Settlement	[100]

? Molecule is not known. C4-HSL—butanoyl-homoserine lactone; C6-HSL—hexanoyl-homoserine lactone; C8-HSL—octanoyl-homoserine lactone; C10-HSL—decanoyl-homoserine lactone; 3-oxo-C10-HSL—3-oxo-decanoyl-homoserine lactone; C12-HSL—dodecanoyl-homoserine lactone; 3-oxo-C12-HSL—3-oxo-dodecanoyl-homoserine lactone.

## Supplementary Materials

Supplementary materials can be found at https://www.mdpi.com/1422-0067/21/3/731/s1.
